# The role of purinergic signaling in the etiology of migraine and novel antimigraine treatment

**DOI:** 10.1007/s11302-015-9453-8

**Published:** 2015-05-10

**Authors:** Marek Cieślak, Joanna Czarnecka, Katarzyna Roszek, Michał Komoszyński

**Affiliations:** 1 Neurological Department, Ludwik Rydygier Voivodship Polyclinical Hospital, 53/59 św. Józefa St, 87-100 Toruń, Poland; 2 Department of Biochemistry, Faculty of Biology and Environment Protection, Nicolaus Copernicus University in Torun, Torun, Poland

**Keywords:** Migraine, Ecto-purines, Purinergic signaling, Vasomotor mechanism, Cortical spreading depression, Trigeminal ganglion

## Abstract

Etiopathogenesis of migraine involves different structures of the central nervous system: the trigeminal nerve with nuclei located in the brain stem, vascular system, and the cerebral cortex as well as diverse mechanisms and pathological processes. The multidirectional action of purines in different cell types (blood vessels, neurons, and satellite glial cells) and through different types of purinergic receptors contributes to the etiopathogenesis of migraine pain. Adenosine triphosphate (ATP) and its derivatives are involved in initiation and propagation of migrenogenic signals in several ways: they participate in vasomotor mechanism, cortical spreading depression, and in fast transmission or cross-excitation based on the satellite glial cells in trigeminal ganglion. Contribution of purinergic signaling in the conduction of pain is realized through the activation of P1 and P2 receptors expressed widely in the central nervous system: on the neurons and glial cells as well as on the smooth muscles and endothelium in the vascular system. Therefore, the purinergic receptors can be an excellent target for pharmacologists constructing new antimigraine therapeutics. Moreover, the mechanisms facilitating ATP and adenosine degradation may prevent vasodilatation and thus avoid a secondary central sensitization during a migraine attack. Thus, agonists and antagonists of P receptors as well as ecto-enzymes metabolizing nucleotides/nucleosides could gain the growing attention as therapeutic agents.

## Introduction

Migraine is defined as recurrent attacks of headache accompanied by autonomic symptoms, with the presence or absence of aura (according to Headache Classification Committee of the International Headache Society, 2013) [1].

Currently, it is known that the etiopathogenesis of migraine involves different brain structures: the trigeminal nerve with nuclei located in the brain stem, vascular system, the cerebral cortex, and diverse mechanisms and pathological processes, involving uncontrolled activation of the trigeminal nerve, vasoconstriction and vasodilatation, and cortical spreading depression [2]. These processes are mediated by pathologically changed concentrations of extracellular signaling molecules and neurotransmitters, and their action effects primarily in the inflammation and pain. There is some evidence that confirms the involvement of purines in the above-mentioned processes throughout the central nervous system [3].

Nucleotides participate in the pathophysiology of migraine through influencing the vasomotor mechanism. Their effects may be contradictory depending on the localization and type of P receptors. Vasoconstriction of blood vessels in the brain induces hypoxia, which stimulates the release of adenosine triphosphate (ATP) from nerve endings to the vascular lumen. ATP together with adenosine are the factors initiating relaxation of the blood vessels which results in an increased cerebral blood flow and is linked with pain [4]. Presently, there are reports that question the participation of vasomotor mechanisms in the initiation of migraine, especially in terms of the large brain arteries [2].

The pivotal process in the initiation of migraine pain is the activation of the trigemino-vascular system [5–8]. This process causes the release of vasoactive molecules and activation of their receptors on vascular smooth muscles [9]. It subsequently results in vascular relaxation, which is associated with pain, an increase in the excitability of primary trigeminal neurons and peripheral or central sensitization (allodynia) [10]. The migrenogenic signals associated with the trigeminal nerve are realized by painful stimuli mediated by fast transmitters (like ATP) or based on the satellite glial cells that mediate the conduction of painful stimuli (e.g., mediated by ecto-nucleotides) deep into the nervous fiber [11].

More and more researchers and clinicians follow the neural-vascular theory, combining initiation, amplification, and propagation of migraine pain not only with vascular reaction but also with the response of cortical neurons. During cortical spreading depression, ATP is released into the brain extracellular space and cerebrospinal fluid which results in the activation and sensitization of afferent sensory fibers of the trigeminal ganglion neurons and the transmission of nociceptive signal [12].

It can be assumed that purinergic receptors can be a target for pharmacologists constructing new antimigraine therapeutics. Moreover, the mechanisms facilitating ATP and adenosine degradation may prevent vasodilatation and thus avoid a secondary central sensitization during the migraine attack. This article is focused on the particular role of purinergic system elements in the pathophysiological processes initiating and amplifying the migraine pain. We also discuss the therapeutic perspectives of purinergic compounds as potential drugs for the acute and preventive antimigraine treatment.

## Purinergic signaling in the central nervous system

The nucleotides and adenosine are released into the extracellular matrix directly from neurons and astrocytes by exocytosis or through a channel or pore. These molecules function as neurotransmitters and neuromodulators that regulate physiological processes such as neurotransmission, proliferation, activation of inflammation, or cell death [13]. Receptors for these compounds are divided into P1 receptors (A1, A2A, A2B, A3) activated by adenosine and P2 receptors activated by nucleotides (ATP, ADP, UTP, UDP). Based on the fundamental differences in their structure and mechanism of signal transduction, the P2 receptors are divided to metabotropic P2Y (1, 2, 4, 6, 11, 12, 13, 14) and ionotropic P2X receptors (1–7), which are described as heteromultimers, e.g., P2X2/3, P2X1/5, and P2X4/6 [14]. Both P1 and P2 receptors are present on most cells throughout the central nervous system [15–17].

Extracellular nucleotide concentration and, thus, nucleotide-mediated signals are controlled by ecto-nucleotidases: ecto-nucleoside triphosphate diphosphohydrolases (ecto-NTPDases), ecto-nucleotide pyrophosphohydrolases/phosphodiesterases (ecto-NPPs), ecto-5′-nucleotidase (5′-NT), and ecto-nucleotide kinases. In the central nervous system (CNS), these enzymes are expressed on the cell surface of astrocytes, oligodendrocytes, microglia, and endothelial cells. The high activity of NTPDase1 and NTPDase2 was observed on the cell membranes of the cerebral cortex and hippocampus, while the activity of these enzymes in the cerebellum and the medulla oblongata is rather low [18]. The final product of the degradation of extracellular ATP and ADP is adenosine, further metabolized with the participation of ecto-adenosine deaminase to inosine. In most areas of the brain, there is a high activity of ecto-5′-nucleotidase and ecto-adenosine deaminase.

It is known that in the CNS, adenine-derivative nucleotides (ATP and ADP) and uracil derivatives (UTP, UDP, and UDP-glucose) are associated with the sensory system and serve as co-transmitters and/or neuromodulators. ATP, in addition to participate in the fast synaptic signaling, affects also the signaling mediated by other neurotransmitters. The correlation between the concentration of ATP and catecholamine release within the locus coeruleus and hippocampus was found [19]. There is also a correlation between the amount of ATP and glutamate released in the hippocampal area [20, 21]. The majority of processes activated by ATP (e.g., secretion of glutamate or propagation of inflammatory reactions) is inhibited by adenosine (which inhibits glutamate release from astrocytes and acts anti-inflammatory) formed during enzymatic degradation of ATP. It indicates that under physiological conditions, there is a balance between ATP-mediated activation and quenching of these processes by adenosine [22].

In 1929, Drury and Szent-Gyorgyi demonstrated for the first time the presence of purines in the blood vessels [23]. The role of purines in the physiology of the nervous system was shown for the first time in 1972 [24], and in the 1980s, Geoffrey Burnstock announced in the journal *Lancet* the hypothesis concerning the participation of purinergic signaling in the pathophysiology of migraine [25, 26]. Based on our recent knowledge about the role of purinergic signaling in brain, the aspect of purinergic drugs represents a promising field for the investigation of novel purinergic migraine treatment strategies.

## The role of ATP and adenosine in the vasomotor etiology of migraine

The widely offered migraine treatment is based on the vasomotor theory, acts primarily on the vascular system, and leads to the constriction of blood vessels within the brain. Triptans are at present the most effective vasoactive drugs terminating a migraine attack, by activation of serotonin receptors 5-HT1B on arterial smooth muscles. They also inhibit the activity of peripheral trigeminal nerve endings by influencing the 5-HT1D or 5-HT1F receptors. Moreover, triptans inhibit neurons in the trigeminal sensory nuclear complex (TSNC) of the brain stem and upper cervical spinal segments by an action on 5-HT1B/5-HT1D/5-HT1F receptors. Unfortunately, many migraineurs do not respond satisfactorily to triptans, and cardiovascular comorbidities (i.e., vasoconstriction in thoracic blood vessels) limit their use in a significant number of patients [27, 28].

**Fig. 1:**
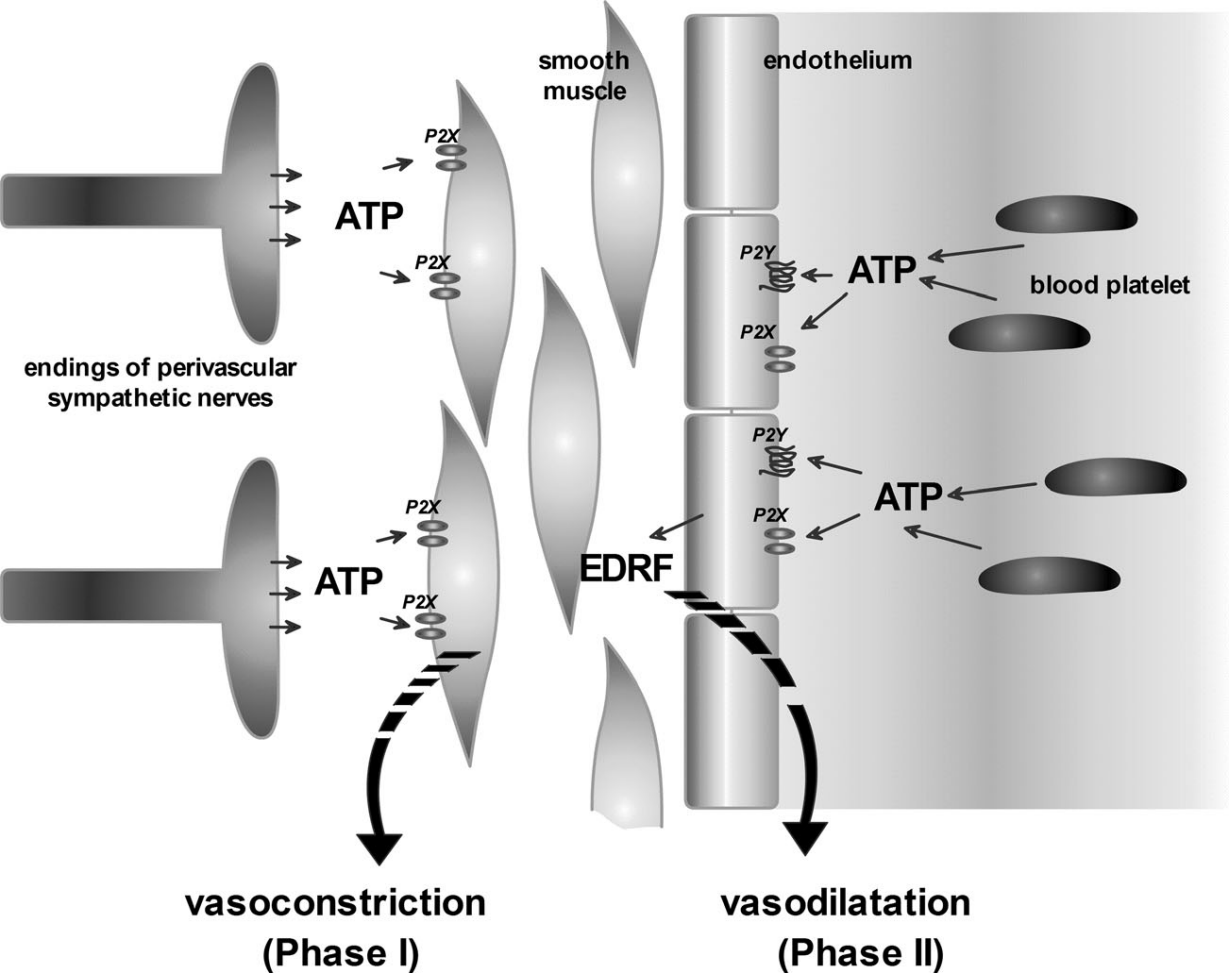
The dual role of ATP involved in vasomotor etiology of migraine attack

The earliest hypothesis for the initiation of migraine pain was the vasomotor theory [29]. This theory assumes that migraine attack consists of two stages. Initially, there is a vasoconstriction (vasospasm), which results in tissue hypoxia but is not accompanied by pain. It is known that local vasoconstriction may be a consequence of activation of P2X receptors present on smooth muscles through ATP released both as co-transmitter with noradrenaline (NA) from perivascular sympathetic nerves and from damaged endothelial cells (Fig. 1). The intracellular stores (sympathetic noradrenaline stores) are depleted of NA in the beginning of a migraine attack, which is correlated with the increased release of co-transmitters and other compounds such as dopamine, prostaglandins, ATP, and adenosine [30]. Vasoconstriction is then followed by vasodilatation which results in reactive hyperemia associated with pain perception [4]. Moreover, the synthesis of ATP and its release from endothelial cells and platelets into blood serum increase significantly during a migraine attack. The released ATP activates the P2X and P2Y receptors (also activated by ADP, UTP, ADP) on the endothelial cells. Their activation initiates the release of endothelium-derived relaxing factor (EDRF) into the blood [3, 4]—Fig. 1. This “purinergic” mechanism explains the dual involvement of purines in the migraine headache initiation.

The cerebral vasculature has been crucial for various hypotheses concerning the pathophysiology of migraine. Malmsjö and collaborators indicate that extracellular nucleotides induce contractions of cerebral arteries primarily by activation of P2Y6 receptors expressed on smooth muscle cells [31]. A recent paper of Haanes and Edvinsson points out that P2X1 and P2Y6 receptors are the strongest contractile receptors in middle meningeal artery (MMA) and therefore regulate the blood flow through the MMA. Adenosine acts as a relaxing factor primarily via endothelial A2A receptors [32]. It has been shown that during a migraine attack, adenosine concentration in the blood increases by about 47 % [33]. Involvement of adenosine in the etiology of migraine pain was confirmed by intravenous administration of adenosine resulting in migraine-like symptoms [34, 35]. Already in 1973, Paalzow and collaborators have found that methylxanthine, a nonselective antagonist of adenosine receptors, reduces the sensory nociceptive threshold in rats [36]. In patients treated with dipyridamole, an increase in migraine attacks was noted, which may be associated with impaired intracellular adenosine uptake from the extracellular space [37]. The observed effect of dipyridamole may, however, be linked to both its inhibitory action on adenosine transporters as well as the inhibition of phosphodiesterases (PDEs) and therefore the increased concentration of cyclic guanosine monophosphate (cGMP). As phosphodiesterase PDE5 is present in platelets and smooth muscles of blood vessels, the inhibition of its activity and consequently an increase in cGMP levels cause effects similar to adenosine: cerebral vasodilatation and initiation of headache [38, 39]. However, the occurrence of headache may not depend so much on the concentration of adenosine itself but on the increasing release of neurotransmitters and other active compounds, such as calcitonin gene-related peptide (CGRP) [40, 41]. CGRP is released from nerve endings following depolarization. The compound is one of the most potent vasodilatory factors throughout the vascular system, especially in the microvasculature. Activation of CGRP receptors on vascular smooth muscles results in their relaxation, which is associated with pain during a migraine attack [42]. Thus, headache may be related to cerebral vasodilation, mainly within the middle cerebral artery, which is responsible for blood supply for the dura mater. Administration of CGRP causes initiation of headache, accompanied by dilation of both the middle meningeal artery and middle cerebral artery [43]. It is worth noticing that activation of G protein-coupled receptors sometimes triggers the phenomenon known as transactivation, which results in synergic or antagonist signaling. In the brain, the action of CGRP is inhibited by A1 receptors and activated by A2A receptors. The ability of A2A receptors to inhibit A1 receptors may also contribute to further facilitation of CGRP action [44].

On the other hand, the adenosine-independent mechanism was confirmed by Hegedus and collaborators, who demonstrated a correlation between the decrease in cerebral blood flow and blood levels of cAMP, but found no similar correlation with the concentration of adenosine [45]. It is now believed that the vasodilation is not sufficient for the activation of the headache, as vasodilation itself is observed, i.e., during decrease in blood pressure.

## ATP and adenosine in trigeminal nerve and satellite glial cell pathophysiology

The trigeminal nerve is the fifth cranial nerve, which largely consists of afferent sensory fibers, and is in the small percentage of the efferent motor fibers [5]. It comprises of three branches: the ophthalmic nerve (V1), jaw nerve (V2), and the mandibular nerve (V3). The key function of the sensory part of the trigeminal nerve is the conduction of stimuli from pain receptors (nociceptors), proprioceptors, mechanoreceptors, and temperature receptors.

Based on the recent knowledge, the key process in the initiation of migraine pain is the activation of the trigemino-vascular system. This process causes the release of vasoactive molecules, such as calcitonin gene-related peptide, substance P, and pro-inflammatory factors, and signaling molecules such as ATP, serotonin, bradykinin, and prostaglandins. All of these compounds affect both neurons and satellite glial cells (SGCs) located in the trigeminal ganglion [9].

Adenosine is involved in the sensitization of trigeminal neurons since it may inhibit this process [28, 46, 47]. There are numerous reports on the potential role of A1 receptor agonists in migraine and cluster headache. It has been demonstrated that selective A1 receptor agonists (GR79236 and GR190178) inhibit the trigemino-vascular system, both within the trigeminal nucleus, as well as by inhibiting the release of CGRP in the vascular system, while not causing the vasoconstriction [48, 49]. Unfortunately, the therapies with A1 receptor agonists are limited by side effects, e.g., bradycardia and hypotension as the most severe of them. The role of the other adenosine receptors (A2A, A2B, A3) in the etiology of migraine is ambiguous. There are only few reports suggesting that adenosine receptors can display anti-nociceptive properties [50–52].

The role of ATP in the etiology of migraine was previously associated only with the vascular theory of disease as described above. Presently, the growing attention is paid to the neuronal dysfunction and transmission of pain via ATP-activated receptors within the trigeminal nerve. Contribution of purinergic signaling in the conduction of pain in terms of P2 receptor activation by ATP is relatively well known in the field of the dorsal root and dorsal spinal cord and peripheral sensory ganglia [15]. Moreover, the presence of P2X3, P2X2, and P2X2/3 receptors, as well as P2Y1, P2Y2, P2Y4, P2Y6 receptors on neuronal cells of the trigeminal ganglion and dorsal ganglia has been shown [14, 15, 53–55]. It was also described that the P2Y1 and P2Y4 receptors present on trigeminal neurons co-localize with the P2X3 receptor [56]. The satellite glial cells expressed a similar set of P2Y receptors: P2Y1, P2Y2, P2Y4, P2Y6 and P2Y12, and P2Y13 (together with P2X2 and P2X2/3) [57, 58].

In vitro studies suggest that activation of P2X3 or P2X2/3 receptors present on afferent trigeminal nerve endings in the dorsal horns of spinal cord causes an increase in the transmission of pain impulses within the trigeminal nerve [59]. Central sensitization of nociceptive neurons in brainstem may be affected by intrathecal application of agonists and antagonists of the P2X receptors [60]. It was also demonstrated that P2X3 receptor plays a key role in trigeminal neuralgia [61]. The increased release of calcitonin gene-derived protein (CGRP) is dependent on activation of the trigemino-vascular system and coexists with a sensitization of P2X3 receptors [62]. This process plays an important role in the further activation of the nerve cells and in the formation of peripheral and central sensitization.

The concept of P2Y receptors participation in the etiology of migraine is relatively new and still unclear [3, 57, 63]. It is believed that the possible effect of P2Y receptor activation is both analgesic and algogenic [58]. P2Y1 receptor activation may inhibit the P2X3 receptor activity in neurons of the dorsal ganglia, suggesting the anti-algogenic role of ATP and ADP. Intrathecal in vivo administration of UTP and UDP, other P2Y receptor agonists, has been shown to have an analgesic effect, probably due to the inhibition of cytokine release from glial cells [64–66]. Some researchers also point out the participation of P2Y6 receptor located in the trigeminal ganglion neurons in migraine etiology [64, 67].

**Fig. 2:**
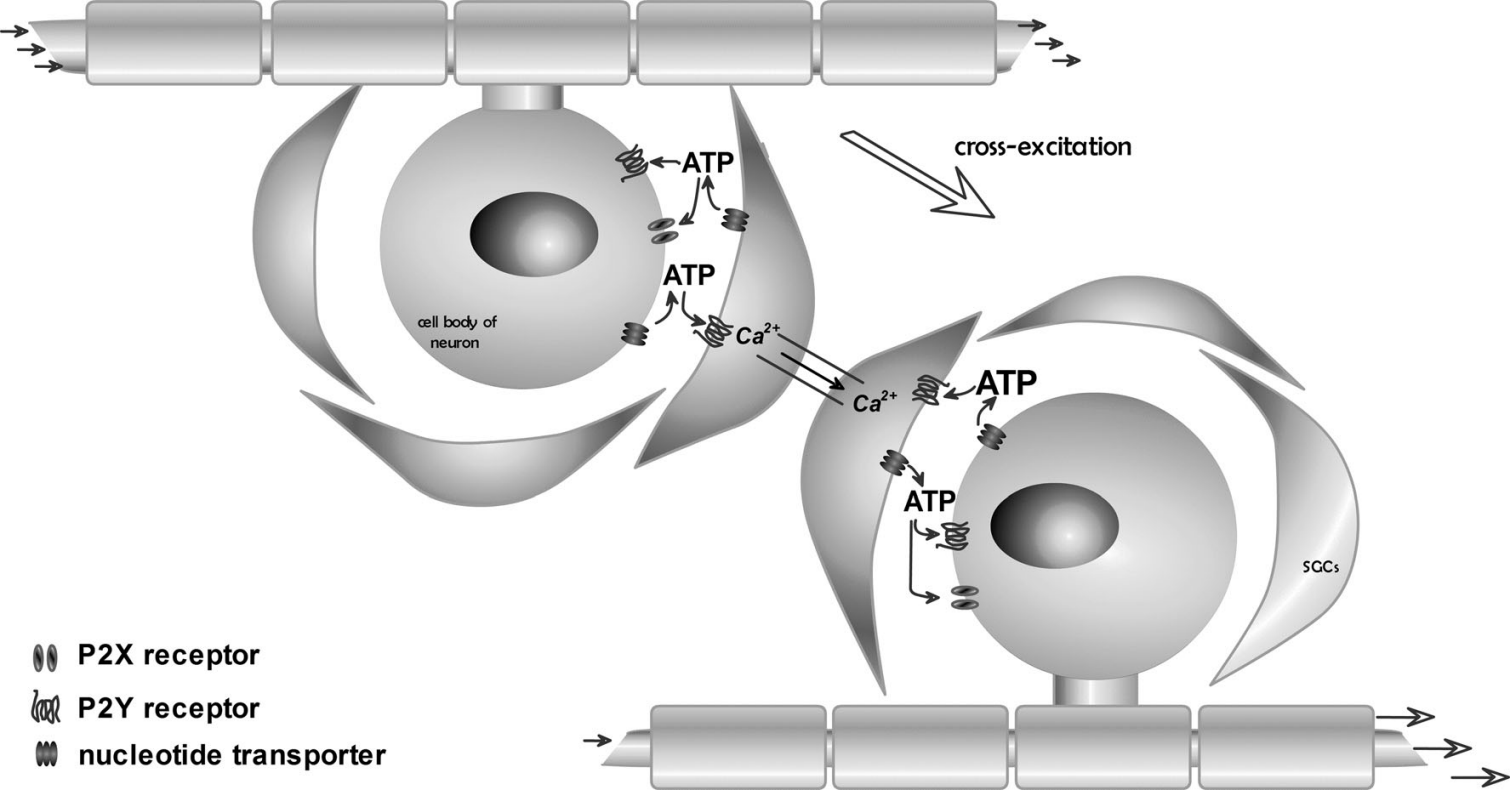
Propagation of pain signal within the trigeminal ganglion (“cross-excitation”). Excitation potentials of neurons cause an increase in ATP release outside the cell. ATP activates P2X receptors on the postsynaptic cell membranes, as well as P2Y receptors on satellite glial cells (SGCs). ATP released from neurons and satellite glial cells causes the spread of Ca^2+^ waves in the neighboring cells, and the excitation spreads out

Satellite glial cells (SGCs), participating in two-way communication between neurons and glia, also enable the propagation of inflammation and pain signals. It is believed that excitation of neurons within the trigeminal ganglion may be expanded not only along the main pathway of nerve impulses. It has been found that nucleotides secreted by trigeminal ganglion neurons increase the calcium concentration inside the satellite glial cells. This means that the satellite glial cells using nucleotide signaling and calcium wave may transfer information into the nervous system in the process called “cross-excitation” [68]—Fig. 2.

Research of Weick and colleagues showed that, despite the large representation of P2 receptors in SGC membranes, pain stimuli are conducted only via P2Y1, P2Y2, and P2Y4 receptor signaling [57]. Neural-glial communication within the trigeminal nerve is significantly weakened by carbenoxolone (a potent blocker of gap junctions) and suramin, P2 receptor antagonists with a broad spectrum of action [69]. That confirms the participation of extracellular nucleotides in the process of neural-glial communication.

The hypothesis concerning participation of the SGCs in the propagation of signals may explain the influence of peripheral nervous system damage on the increase of the excitability of many sensory neurons that can cause chronic pain [70]. Results of Ceruti and colleagues, who used bradykinin (BK) as an activator of sensory neurons, may expand this hypothesis with the factors that cause sensitization of the sensory system and link it to migraine headaches [8]. In the trigeminal ganglion neuronal cultures, it was found that BK as an algogenic mediator sensitizes the P2X3 receptors, which increases impulse conduction to the trigeminal nuclei in the brainstem and the conduction of painful stimuli in migraine. This process is possible by changing the expression and function of P2X receptors initially through an increased trafficking of the receptor, followed by the increase in its synthesis [71]. It was also shown that this receptor sensitivity may be affected by other mediators such as CGRP, NGF, and BDNF [64, 72–74]. In the case of P2Y receptors, bradykinin causes increased expression and increased sensitization of these receptors. Further, it causes an increase in the release of CGRP from trigeminal neurons, which can also sensitize P2X3 receptor and enhance functions of P2Y receptors on satellite glial cells [58, 71, 74, 75].

The increase in purinergic sensitivity of satellite glial cells is accompanied by a significant increase in the release of pro- and/or anti-inflammatory cytokines that can influence the interaction between the cells in the trigeminal ganglion [76, 77]. The results of Takeda and colleagues suggest that activation of the satellite glial cells modulates neuronal excitability within trigeminal ganglion through the IL-1β released in the inflammatory process. At the same time, an increase in the IL-1β receptor activity in the course of inflammation may contribute to the occurrence of hyperalgesia [77]. The most extreme consequence of hypersensitivity is allodynia, when a sensation of pain is due to a stimulus that normally causes no pain. This phenomenon is accompanied by the neurogenic inflammation in the brain meninges. Evidence for the existence of central sensitization (also referred to as skin allodynia in the area of innervation by the trigeminal nerve) emerges from an analysis of patients with migraine interviews. Some of them are complaining about the sensitivity of the scalp to the small tactile stimuli during daily activities such as washing the face, shaving, and combing the hair. Otherwise, there is an activation of inflammatory processes, manifested in the production and release of nucleotides and prostaglandins. The easiest way to break the migraine attack at the stage of neurogenic inflammation in the meninges is using triptans or ergotamine to prevent the development of a secondary central sensitization [78]. On the other hand, both blocking of the IL-1β receptor, as well as modulation of nucleotide signaling, prevent from pro-inflammatory interleukin secretion and may become potential drugs to avoid hyperalgesia. It will be also effective in the inhibition of another mechanism of neuronal sensitization mediated by chronic trophic ATP influence and neuronal plasticity [79, 80].

## Cortical spreading depression

The aura is a set of specific symptoms and warning signs that precede the migraine headache. This phenomenon occurs in about 30% of patients with migraine [81–83]. The dominant cause of the aura is the phenomenon of cortical spreading depression (CSD), occurring 30–60 min before the migraine headache attack [84, 85]. CSD consists of hypoperfusion waves slowly spreading in the cerebral cortex, accompanied by the wave of depolarization and the lack of neuronal activity, with subsequent activation of the trigemino-vascular system [86, 87]. A wave of depolarization of neurons is preceded by vasodilatation of cerebral vessels, both small and larger ones [88].

It is presently well known that migraine headache is preceded by activation of nociceptive receptors in the meninges and subsequent activation of the sensory fibers of trigeminal neurons that innervate the blood vessels within the meninges [89]. Recently, Zhang and colleagues demonstrated for the first time that CSD activates not only the primary neurons of the trigeminal ganglion but also the secondary neurons located in the upper segments (C1–C2) of the spinal cord [90]. Such molecules as ATP, nitric oxide, prostaglandins, and potassium ions (K^+^) are released into the cerebrospinal fluid during cortical spreading depression [91, 92]. The increase in the concentration of these molecules results in the activation and sensitization of afferent sensory fibers of the trigeminal ganglion neurons and the transmission of nociceptive stimuli from meninges to secondary neurons of the trigeminal nerve (trigeminal sensory nuclear complex, TSNC) located in the brain stem and upper cervical spinal segments. Already in the period of increased activity of the cerebral cortex that is directly followed by CSD, there is a significant increase in the concentration of ATP in the intercellular space. ATP is responsible for the activation and amplification of nociceptive signal transmission and inflammation, and it consequently leads to the activation and sensitization of afferent sensory fibers [91, 92]. The nociceptive and proinflammatory effect of ATP is triggered by the activation of P2X and P2Y receptors, present on the primary afferent fibers, primarily on the nociceptive fibers C [3, 15, 93–95].

Most of the nociceptive trigeminal neurons, that innervate the dura, express P2X3 receptors and are capable of releasing calcitonin gene-derived protein (CGRP) [14, 66]. P2X3 receptor activation facilitates the release of CGRP within the dura mater and promotes the initiation of inflammatory processes, as well as the further release of CGRP in the trigeminal nuclei of the brainstem and, consequently, causes sensitization of secondary neurons involved in the transmission of painful stimuli [96]. Moreover, CGRP causes sensitization of nociceptive receptor P2X3 present in the trigeminal ganglion [66]. Studies of Masterson and collaborators showed that dihydroergotamine (DHE), through the activation of α(2)-adrenoreceptors, blocks ATP-induced sensitization of trigeminal neurons, inhibits the release of CGRP, and reduces the expression of membrane receptor P2X3 [96].

## Therapeutic perspectives

Despite the complexity of migraine pathophysiology, substantial advances have been achieved over the past 20 years in its understanding and development of pharmacological treatment. Triptans, agents with the vasoconstrictor activity, are currently the most effective drugs in the interruption of a migraine attack. However, there is a significant need for novel therapeutic drugs for the acute and preventive treatment of migraine [97]. Based on the experimental evidence presented, it has become increasingly apparent that the purinergic system significantly contributes to nociceptive signaling. The multidirectional action of purines in different cell types (blood vessels, neurons, and satellite glial cells) and through different types of purinergic receptors contributes to the initiation and amplification of migraine pain. Therefore, the purinergic receptors can be an excellent target for pharmacologists constructing new antimigraine therapeutics [98]. Adenosine has been reported to trigger migraine attack while dipyridamole, an adenosine uptake inhibitor, can increase migraine attack frequency. Therefore, A1 receptor stimulation has been already proposed for migraine treatment [99]. Unfortunately, a significant limitation of use of A1 receptor agonists is their side effects outside the central nervous system as described above. Results of recent studies indicate that acute, long-lasting sensitization of trigeminal nociceptive neurons occurs via enhanced expression of ATP-gated P2X3 receptors [73]. The P2X3 receptor is the only ligand-gated channel known to be expressed exclusively by a subset of trigeminal and spinal sensory neurons and may be a promising candidate for antimigraine drug development [100]. The problem of developing migraine treatment based on purinergic signaling is due to insufficient knowledge about the effects of activation of P2X receptors and P2Y present in the trigeminal ganglion neurons and satellite glial cells by adenine nucleotides (ATP and ADP) and uracil derivatives (UTP, UDP, and UDP glucose). Because it is believed that the effect of these nucleotides is algogenic, it is possible to achieve analgesic effect with intravenous or intrathecal application of P2X and P2Y receptors antagonists. The studies on highly specific, non-nucleotide P2X3 and P2X2/3 receptor antagonists, such as A-317491 and AF-219, were already published [101–103]. AF-219 was confirmed as an excellent medicinal candidate to establish the role of P2X3 receptors in chronic pain and related conditions. The compound was particularly efficacious in rodent models of hyperalgesia (neuropathic and inflammatory) or visceral hypersensitivity [103]. The favorable pharmacodynamic and pharmacokinetic properties as well as in vivo activity of AF-219 result in its further exploration in clinical trials.

As seen from clinical trials, the current therapeutic strategies for CNS disorders, including migraine, focus rather on the use of P1 and P2 receptor antagonists. It is important to note that most of the analyzed receptor antagonists partially inhibit nucleotidase activity, so they indirectly participate in maintaining high ATP concentration. Ecto-NTPDase1 knockout augmented purinergic vasorelaxation in vitro and the hypotensive effects of purines in vivo [104]. We postulate that upregulation of ATP metabolism in the local environment of cells through ecto-NTPDase activation or delivery may provide the best therapeutic solution [105]. The mechanisms facilitating ATP and adenosine degradation may prevent vasodilatation and thus avoid a secondary central sensitization during a migraine attack. Therefore, ecto-enzymes metabolizing nucleotides/nucleosides could gain growing attention as therapeutic agents. The complexity of the purinergic signaling system poses several limitations, especially in the translation to the clinic. Limited evidence is available on the spatiotemporal characterization of the purinergic effects in the trigeminal vascular system in vivo. These issues are complicated by the lack of an appropriate animal model representative of complex migraine pathology. However, we should not forget that, in the future, drugs for migraine may be ecto-enzymes themselves or compounds that modulate their activity.

## Summary

No hypothesis has yet been proved capable of explaining all the features of migraine headache. However, the purinergic signaling system gains the growing interest in terms of its involvement in migraine etiology as well as in its potential therapeutic importance. ATP and its derivatives are involved in initiation and propagation of migrenogenic signals in several ways: they participate in vasomotor mechanism, cortical spreading depression, and in fast transmission or cross-excitation based on the satellite glial cells in trigeminal ganglion.

ATP is involved in vasoconstriction and vasodilatation, and the latter is accompanied by the occurrence of pain. The vasoconstrictory effect of ATP originates from the local activation of the P2X receptors present on smooth muscles. The coaction of ATP and other compounds such as noradrenaline, dopamine, and prostaglandins is responsible for effective vasoconstriction. The ATP involvement in the process of the vasodilatation effects in activation of endothelial P2X and P2Y receptors, followed by the release of the endothelium-derived relaxing factor (EDRF) into blood. It is also believed that the increase in the concentration of adenosine in the extracellular space, including the blood, may trigger vasodilatation and thereby cause headache.

During cortical spreading depression, there is a significant increase in the concentration of ATP in the intercellular space followed by its release to the cerebrospinal fluid. The elevated concentration of ATP and other active molecules results in the activation and sensitization of afferent sensory fibers of the trigeminal ganglion neurons and transmission of nociceptive information from the meninges to the secondary trigeminal neurons via P2X3 receptor.

The presence of P2X and P2Y purinoreceptors on trigeminal ganglion neurons and glial satellite cells, as well as the discovery of their participation in the conduction of nociceptive stimuli, confirms the role of nucleotides in the etiopathogenesis of migraine. The presence of the A1 receptors on trigeminal neurons suggests that adenosine is also involved in the sensitization of these neurons.

The multidirectional action of purines in different cell types (blood vessels, neurons, and satellite glial cells) and through different types of purinergic receptors contributes to the initiation and amplification of migraine pain. Therefore, the purinergic receptors can be an excellent target for pharmacologists constructing new antimigraine therapeutics. It can be also assumed that the mechanisms facilitating ATP and adenosine degradation may prevent vasodilatation and thus avoid a secondary central sensitization during the migraine attack.
